# Superagonistic CD28 protects against renal ischemia injury induced fibrosis through a regulatory T-cell expansion dependent mechanism

**DOI:** 10.1186/s12882-019-1581-x

**Published:** 2019-11-09

**Authors:** Yiran Liang, Ning Xue, Xiaoyan Wang, Xiaoqiang Ding, Yi Fang

**Affiliations:** 1 Department of Nephrology, Zhongshan Hospital, Fudan University, 111 Yixueyuan Road, Shanghai, 200032 China; 2 Shanghai Medical Center of Kidney, Shanghai, China; 3 Shanghai Key Laboratory of Kidney and Blood Purification, Shanghai, China; 4 Shanghai Institute of Kidney and Dialysis, Shanghai, China

**Keywords:** Regulatory T cell, CD28 superagonists, Acute kidney injury, chronic kidney disease

## Background

Acute kidney injury (AKI) affects almost 5% of all hospitalizations, with high morbidity and mortality [1]. The long-term prognosis of AKI is extremely poor. A 10-year follow-up study of AKI patients found that 19 to 31% of AKI patients eventually progressed to chronic kidney disease (CKD) or end stage renal disease (ESRD), and 12.5% of the patients required long-term dialysis [2–4]. The mechanisms driving the AKI to CKD/ESRD transition remain unclear. Inflammatory immune response plays an important role in AKI pathogenesis and has recently attracted attention [5–8]. CD4^+^ T cells differentiate into various effector T-helper (Th) cells upon antigen exposure and cytokines in the post-ischemic milieu. Th1 cells promote acute kidney injury whereas Th2 cells contribute to tissue repair. Th17 cells identified by interleukin-17(IL-17) secretion, have been verified to persist in the kidney for up to 5 weeks following initial acute kidney injury [9]. Regulatory T cells (Tregs), another Th cells subset, promote recovery from renal ischemic injury in an interleukin-10 (IL-10) dependent pathway [10].

Renal ischemia-reperfusion injury (IRI) is regarded as acute inflammatory disease, whereas immune cells play crucial roles during the entire process of AKI, extending across initiation, maintenance, and recovery phases [11]. Dendritic cells and macrophages demonstrate a paranoid effect when differentiating into various phenotypes. Treg-mediated negative immune-regulation was proven in various experimental models wherein Tregs were stimulated by different reagents such as interleukin-2(IL-2) /anti-IL-2 [12], IL-17A [13] and superagonistic CD28 (CD28sa), which strongly supports the protective role of Tregs against over-activation of inflammation [14].

In our previous study, we proved that CD28sa pretreatment as a pre-ischemia intervention against IRI, needs about 6 days to work at full capacity in order to stimulate an adequate amount of Tregs [14]. We were then interested in the role of Treg expansion during the AKI to CKD transition. Therefore, we designed the present study to investigate the renal chronic outcome at as long as 28 days after IRI injury following CD28sa pretreatment.

## Methods

### Experimental animals and surgical protocol

Six to eight-week-old male C57BL/6 J mice (weight, 20–25 g) were purchased from Silaike (China), 8–10 for each subgroup. Animals were housed in temperature- and humidity-controlled cages, with free access to water and rodent food on a 12-h light/dark cycle. Experiments were completed at Zhongshan Hospital Fudan University. The animal use protocols were approved by the Institutional Animal Care and Use Committee of Fudan University and Zhongshan Hospital. They also strictly adhered to the National Institutes of Health Guide for the Care and Use of Laboratory Animals. All surgeries were performed under intraperitoneal 1% sodium pentobarbital anesthesia (3.0–4.5 ml/kg), intrarectal temperature of mice was maintained at 35.0 °C–36.0 °C with a heating pad during the surgeries. A midline abdominal incision was made, and the mice were subjected to bilateral renal pedicle clamping for 35 min using non-traumatic microvascular clips. After removal of the clamps, blood restoration in the kidneys was confirmed visually. To maintain body fluid balance, all mice were supplemented with 0.5 ml of saline administered subcutaneously. A sham operation was performed in a similar manner, except for renal pedicle clamping. After surgery, mice were transferred to the recovery cages, one cage for each. Parameters like vital signs, time to wake up from anesthesia were monitored. Food intake was daily monitored, and animals were weighed once every other day. Mice were sacrificed at 1, 3, 7, 14, and 28 days after the procedure, and serum and kidney tissues were collected for various analyses. One percent pentobarbital sodium (4 ml/kg) were injected intraperitoneally, 15 min later, mice were sacrificed by cervical dislocation. The early mortality rate (mice died before the indicated time point) of mice subject to IR, ranged from 10 to 30%.

### Protocols of CD28sa administration and Treg depletion

**Fig. 1:**
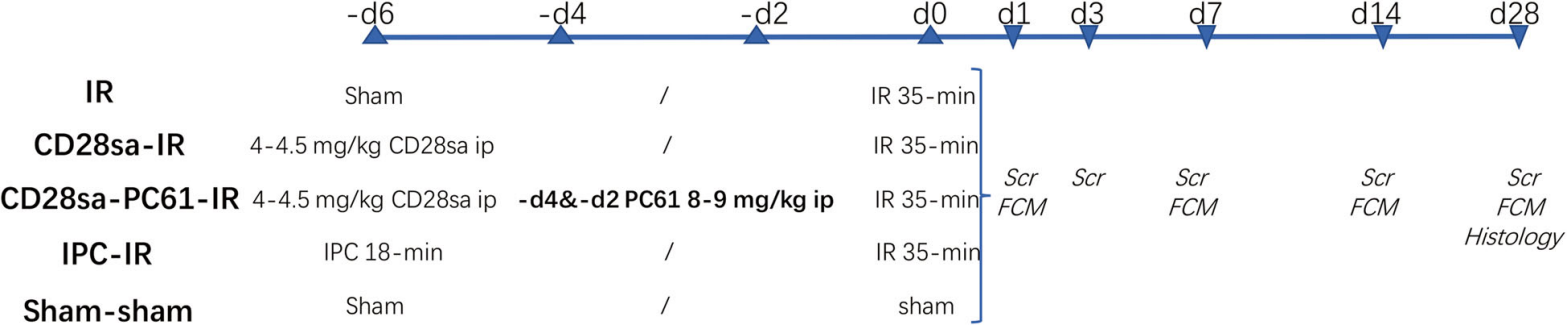
The experiment procedure and group division. IR (35-min ischemia) was induced by bilateral renal pedicles clamping followed by reperfusion. IPC was induced 6 days before IR for 18-min pre-ischemia. For the CD28sa-IR group, a bolus injection of 4–4.5 mg/kg CD28sa in 1 ml phosphate buffer (PBS) was administered intraperitoneally 6 days before IR. Apart from the IR procedure and CD28sa injection, 8–9 mg/kg of monoclonal anti-CD25 antibody (clone PC61, Biolegend, USA) was administered via peritoneal injection for the CD28sa-PC61-IR group on days 3 and 5 following the 35-min ischemia on day 7, for in vivo depletion of Tregs. IR, ischemia reperfusion; IPC, ischemia preconditioning; CD28sa, CD28 superagonists. Scr, serum creatinine; FCM, flow cytometry

Superagonistic CD28 was purchased from AbD Serotec (USA). LEAF-purified anti-mouse CD25 antibody was purchased from Biolegend (USA). For the CD28sa-IR group, a bolus injection of 4–4.5 mg/kg CD28sa in 1 ml phosphate buffer (PBS) was intraperitoneally administered to mice 6 days (day 1) before IR. The ischemia preconditioning (IPC) was induced 6 days before IR for 18-min pre-ischemia. The sham operation group and PBS group were used as controls. In order to deplete Tregs, anti-CD25 antibodies (PC61, Biolegend, USA) were administrated intraperitoneally to mice at a dose of 8–9 mg/kg on day 3 and day 5 for the CD28sa-PC61-IR group as reported in our previous study [14]. On day 7, mice were subjected to IRI injury. Blood, kidney, and spleen tissues were harvested at 24 h, 3 days, 7 days, 14 days, and 28 days after renal IRI. At indicated time point, 1% pentobarbital sodium 4 ml/kg were injected intraperitoneally, 15 min later, mice were sacrificed by cervical dislocation (Fig. 1).

### Immunohistochemistry staining

The number of stain positive cells was analyzed using immunohistochemistry (IHC) as described previously [14]. Anti-Fibronectin rabbit antibody (Cell signaling technology, USA), anti-Collagen IV rabbit antibody (Abcam, USA), and anti-8-OHdg rabbit antibody (Abcam, USA) were used as primary antibodies according to the manufacturer’s instructions. Secondary antibodies (1:5000; Jackson ImmunoResearch) were also used according to the manufacturer’s instructions. All immunohistochemistry staining was analyzed as follows: under the microscope (200x or 400x), the sections were moved randomly to take 20–25 pictures per section, followed by analysis using Image Pro-Plus 6.0 Software (Media Cybernetics, USA) to identify the target cells.

### Fluorescence-activated cell sorting (FACS) analyses

For detection of CD4^+^Foxp3^+^ Tregs, spleens, blood, and kidneys of C57BL/6 mice were subjected to flow cytometry analysis as described previously [14]. Anti-CD4- fluorescein isothiocyanate (FITC), anti-CD25-Phycoerythrin (PE), anti-Foxp3-efluor450, anti-IL-17A-allophycocyanin (APC), anti-CD11c-PE, anti-MHCII-FITC antibodies were purchased from eBioscience (USA). FACS Attune Nxt (Life, Thermo) was used for the analysis.

### Western blot analyses

Relative protein abundances were detected using western blotting as we previously described [6–8, 14]. The antibodies we used in this study were from CST, Abcam, BioLegend and eBioscience.

### Histologic analyses

Renal tubulointerstitial fibrosis was detected using Masson’s trichrome staining or sirius red staining, the extent of renal fibrosis was shown as the percentage of blue-stained area or sirius red-stained area in renal cortex and outer medulla.

### Renal lysate cytokines measurement

The mouse cytokines interleukin-1α(IL-1α/β), IL-2, IL-3, IL-4, IL-5, IL-6, IL-9, IL-10, IL-12 (p40), IL-12 (p70), IL-13, IL-17A, Eotaxin, granulocyte-macrophage colony stimulating factor (GM-CSF), granulocyte-colony stimulating factor (G-CSF), interferon-γ (IFN-γ), keratinocyte chemoattractant, monocyte chemotactic protein-1 (MCP-1, MCAF), macrophage inflammatory protein-1α (MIP-1α), macrophage inflammatory protein-1β (MIP-1β), RANTES, and tumor necrosis factor-α (TNF-α) were quantified using Mouse 23-plex Multi-Analyte Kit (Bio-Plex Suspension Array System; Bio-Rad, Hercules, CA, USA). The antibody array experiment was performed according to their established protocol by Wayen Biotechnology (Shanghai, China). The exact protocol was administered according to what had been reported before [15] .

### Statistical analysis

Data were shown as mean ± standard error. When comparing two samples, student’s t unpaired t test was used. When comparing more than two samples, Dunnet’s post hoc test for parametric data or the Kruskal-Wallist for nonparametric data was used. A *P* value less than 0.05 was considered to be statistically significant. All statistical analyses were done using SPSS software version 17.0.

## Results

### CD28sa administration alleviated IRI-induced renal inflammation

**Fig. 2:**
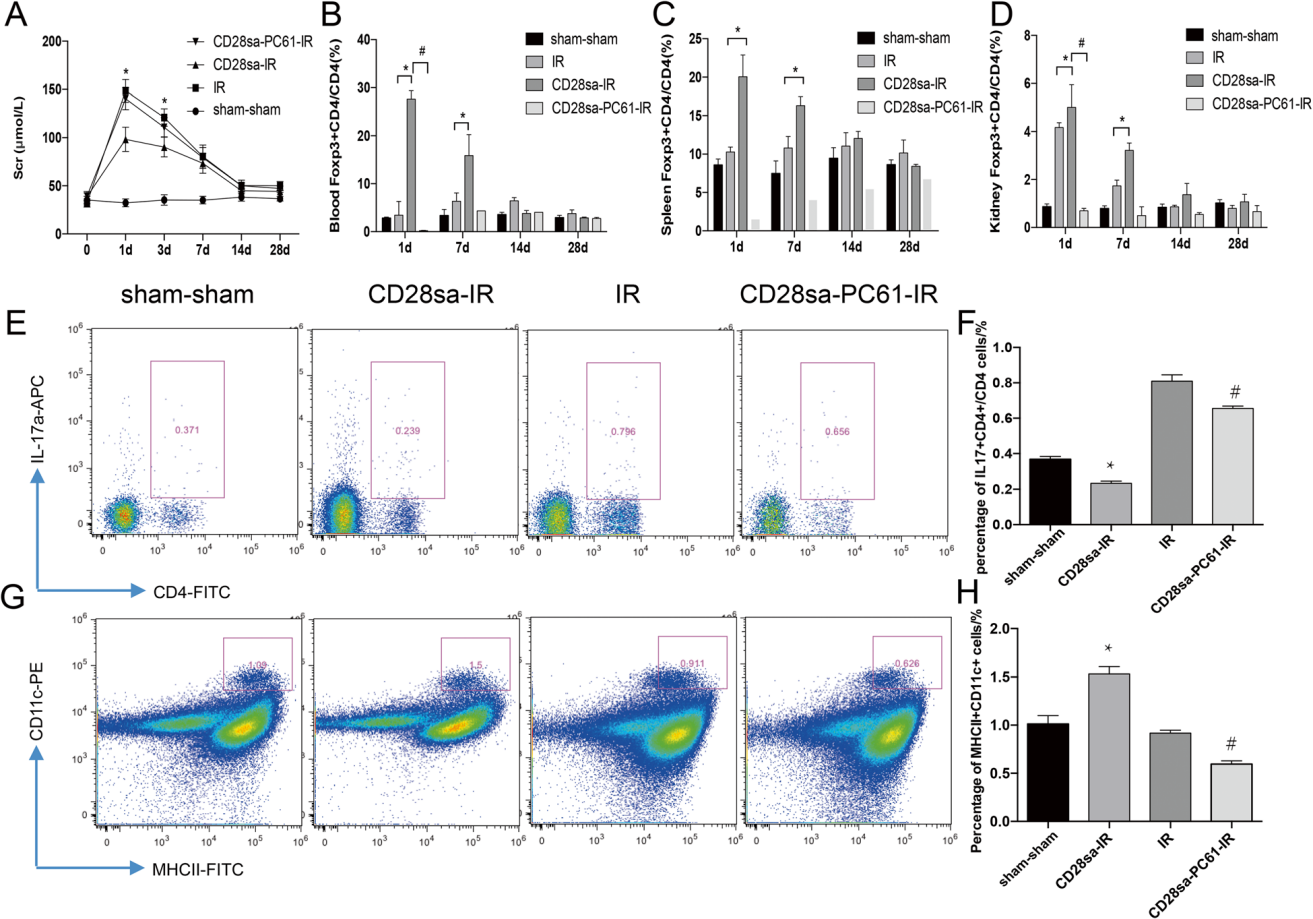
CD28sa promotes Tregs expansion and alleviates renal ischemic reperfusion injury. **a.** Serum creatinine (Scr) was significantly decreased in CD28sa treated mice from day 1 to day 3 post IRI. ^*^P < 0.05, compared with the IR group (*n* = 6). **b-d.** Mice were sacrificed on day 7, 14, and 28 post-ischemia. Mononuclear cells from the peripheral blood, spleen, and kidney were obtained for various analyses. Flow cytometry was applied for the detection of CD4^+^ Foxp3^+^ Tregs and the percentage of Tregs from the total number of CD4^+^ T cells. The percentage of Tregs from peripheral blood, spleen, and kidney was much higher in the CD28sa pre-treated mice than that in the IR group from day 1 to day 7 post IRI. **e.** Flow cytometric detection of IL-17A^+^CD4^+^ T cells in kidneys. **f.** Quantitative analysis of Th17 cells in kidneys. **g.** Flow-cytometric detection of CD11c^+^MHCII^+^ dendritic cells in kidneys. **h.** Quantitative statistics of renal dendritic cells. ^*^P < 0.05 compared with the IR group (*n* = 6). ^#^*P* < 0.05 compared with the CD28sa-IR group (*n* = 6)

When renal function was assessed after IRI, significant improvement was found in the CD28sa group compared to that in the IR group (Fig. 2a). Due to its ability to systemically neutralize CD28sa induced Treg expansion, PC61 abrogated the renoprotective effects of CD28sa on day 1 and day 7 post-IRI (Fig. 2a).

We measured the percentage of Tregs, dendritic cells, and Th17 cells in the kidney, peripheral blood, and spleens by flow cytometry at 24 h, 7 days, 14 days, and 28 days post-IRI in the mice model. Since we previously found Treg expansion reached a peak at 6 days after CD28sa treatment [14], we administered CD28sa or PBS at 6 days before IRI. CD28sa induced a significant increase in the percentage of Foxp3^+^CD4^+^ Tregs of CD4^+^ T cells from the spleen, blood, and kidney (Fig. 2b-d). When anti-CD25 antibody (PC61) was administered after injection of CD28sa on day 3 and 5, expansion of Tregs in the spleen, peripheral blood, and kidney was mostly abrogated in the short term (Fig. 2b-d).

The balance between Th17 and Tregs plays a key role in the maintenance of immune homeostasis in vivo. To illustrate whether the expansion effect of CD28sa could be connected to Th17 cells during IRI-induced inflammation and fibrosis, we checked the Th17 cell percentage at different time points after IRI. The percentage of IL-17A^+^CD4^+^ Th17 cells of the renal tissue indicated a remarkable decrease in the CD28sa-IR group compared with the IR group at 24 h after IRI (Fig. 2e-f). Tregs are capable of inhibiting Th17 cells and other effector T cells. The percentage of CD11c^+^MHCII^+^ dendritic cells in the kidney increased significantly in the CD28sa-ischemia reperfusion (IR) group (Fig. 2g-h). These results suggested that CD28sa treatment inhibited Th17 cell accumulation and promoted Tregs and CD11c^+^MHCII^+^ dendritic cell accumulation in the early stage of post-IRI inflammation.

### CD28sa administration alleviated the IRI-induced renal fibrosis

**Fig. 3:**
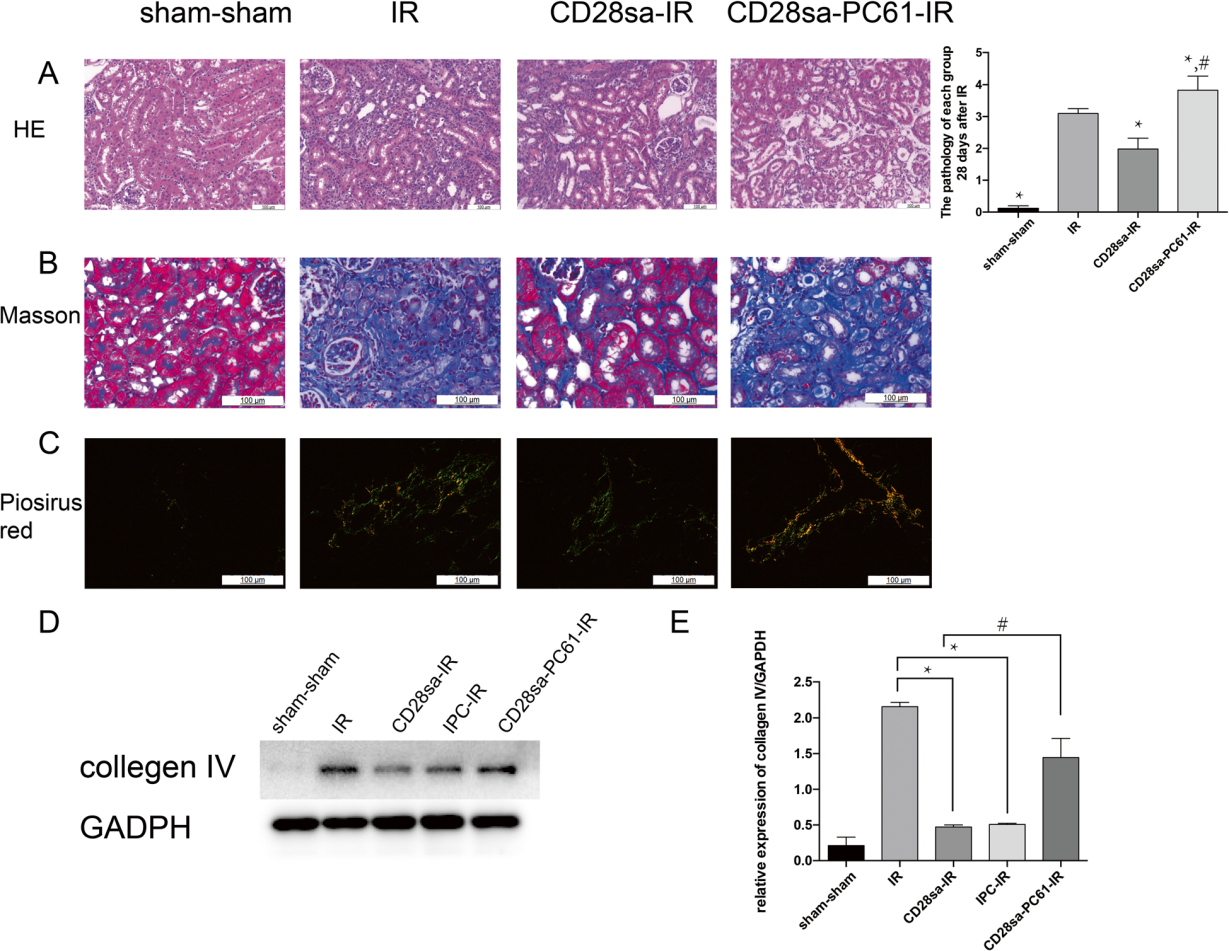
CD28sa pretreatment retards CKD progression post-IRI. Following CD28sa or PC61 pretreatment 6 days before, an ischemia reperfusion injury was performed on day 0 and animals were killed for various analyses on day 7, 14, and 28. **a.** Representative images of hematoxylin-eosin (HE) stained kidney sections (original magnification × 200, Bar = 100 μM). **b.** Representative images of Masson-stained kidney sections (original magnification × 200, Bar = 100 μM). **c.** Representative images of Picosirus-red stained kidney sections (original magnification × 200, Bar = 100 μM). **d.** Immunoblot showed that CD28sa pretreatment significantly downregulated collagen IV expression of the kidneys at 28 days after IRI. **e.** Histogram represented the protein expression of Collage IV in mouse kidneys. These results were from 3 independent experiments, expressed as means ± SEM. ^*^P < 0.05 compared with IR group (n = 6). ^#^*P* < 0.05 compared with the CD28sa-IR group (n = 6)

To further verify the protective effects of CD28sa against IRI-induced renal fibrosis, we checked the histological changes of mouse kidneys. Tubular cell vacuolization, cast formation, and loss of brush border was predominant at the cortico-medullary junction by 28 days after IRI. In contrast, mice treated with CD28sa presented mild renal morphologic abnormalities (Fig. 3a).

Subsequently, we assessed kidney fibrosis at 28 days post-injury. Pathological examination showed no renal fibrotic lesions in the PBS-treated group. Tubulointerstitial fibrosis was prominent in the IRI group, with excess collagen deposition evidenced by masson staining and sirius red staining (Fig. 3b-c). Simultaneously, CD28sa-treated mice showed attenuated renal pathological damage and less collagen deposition (Fig. 3b-c). Western blot also showed that renal expression of collagen IV protein was reduced in the CD28sa-IR group compared with that in the IR group (Fig. 3d-e). As we previously reported, CD28sa mimicked the renoprotective effects of IPC on acute kidney ischemic injury. In this study, we have also observed that CD28sa mimicked the renoprotective effects of IPC on the long-term outcome. As shown in Fig. 3d-e, either IPC treatment or CD28sa treatment significantly attenuated renal protein expression of collagen IV at day 28 post IRI. Conversely, PC61 abolished all the beneficial effects conferred by CD28sa treatment.

### CD28sa attenuated the IRI-induced extracellular matrix deposition and oxidative stress

**Fig. 4:**
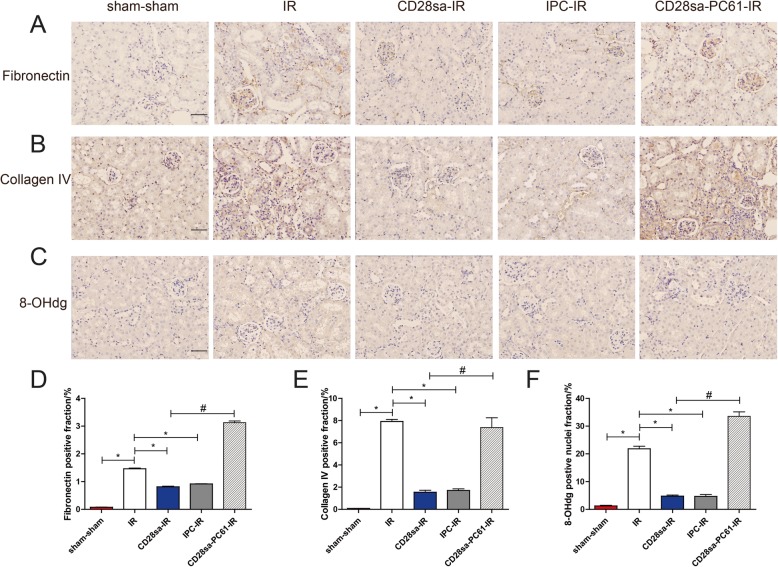
Effects of CD28sa on extracellular matrix deposition and oxidative stress in the kidneys. **a**. Immunochemistry for fibronectin (original magnification × 200, Bar = 100 μM). **b**. Immunochemistry for collagen IV (original magnification × 200, Bar = 100 μM). **c**. Immunochemistry for 8-OHdg (original magnification × 200, Bar = 100 μM). **d**-**f**. Quantitative statistics of fibronectin, 8-OHdg, and collagen IV deposition proportions in the kidney sections of different groups. ^*^P < 0.05 compared with IR group (n = 6). ^#^*P* < 0.05 compared with CD28sa-IR group (n = 6)

Extracellular matrix (ECM) is a three-dimensional network of extracellular macromolecules such as collagen, enzymes, and glycoproteins that provide biochemical and structural support of surrounding cells. We examined the expression of fibronectin and collagen IV to fully indicate the ECM deposition of kidneys. Immunochemistry staining showed that fibronectin and collagen IV deposition induced by IR injury was significantly mitigated by CD28sa treatment (Fig. 4a-b).

Oxidative stress is known to have detrimental effects on T cells and natural killer (NK) cells both in chronic inflammatory conditions and in cancer [16]. Tregs have been reported to possess anti-oxidative capacity in the cancer microenvironment [17], therefore, we checked the expression of DNA associated oxidative stress marker, 8-Oxoguanine (8-OHdg) among different groups. Immunochemistry staining revealed that CD28sa treatment significantly downregulated 8-OHdg expression at day 28 post IRI (Fig. 4c), whereas depletion of Tregs by the anti-CD25 antibody PC61 significantly reversed the anti-oxidative effects secondary to CD28sa treatment. What’s more, CD28sa treatment showed similar anti-fibrotic and anti-oxidative effects with IPC treatment, as both CD28sa-IR group and IPC-IR group presented less fibronectin and Collagen IV deposition as well as less 8-OHdg expression, compared to the IR group (Fig. 4d-f).

**Fig. 5:**
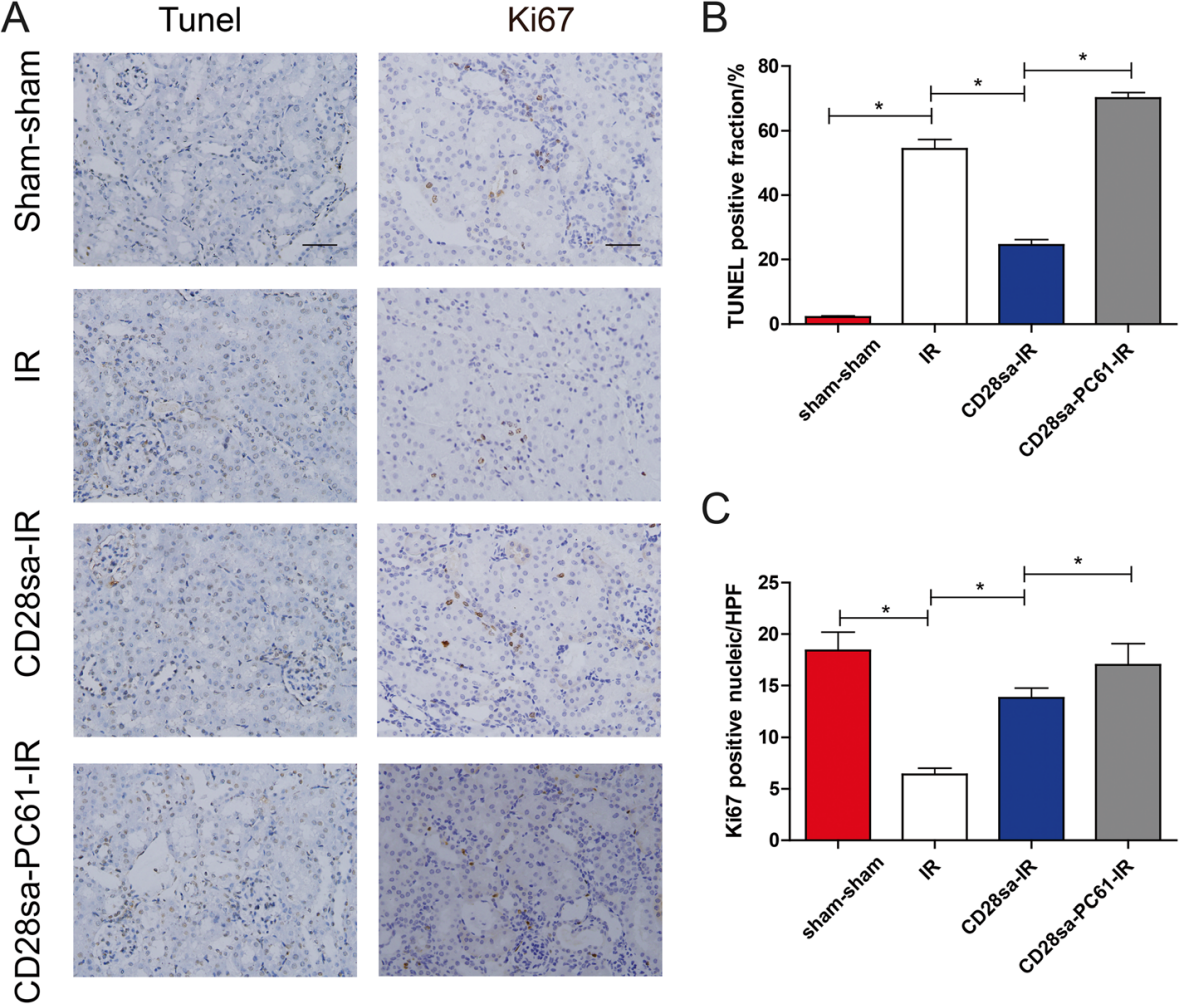
CD28sa mitigated cell apoptosis and proliferation. **a**. TdT-mediated dNTP nick end labeling (TUNEL)-positive cells and Ki67-positive cells of the kidney sections (original magnification × 200, Bar = 100 μM). **b**. Quantification statistics of TUNEL-positive stained cells. **C**. Quantification of Ki67 positive stained nuclei. ^*^P < 0.05 (*n* = 6)

Apoptosis and cell proliferation play a key role in tissue injury and repair [18]. At 28 days post injury, there was still an increased number of TUNEL positive cells in the kidneys of the IR group compared with those in the control group, which was attenuated by CD28sa treatment (Fig. 5a-b). Meanwhile, Ki67 staining of kidney specimens showed an increased number of proliferating cells in mice with CD28sa treatment (Fig. 5a,c). Similarly, these protective effects could be reversed by PC61 administration.

### CD28sa may decrease Th17 cells by inhibiting the expression of IL-6 and IL-17A

**Fig. 6:**
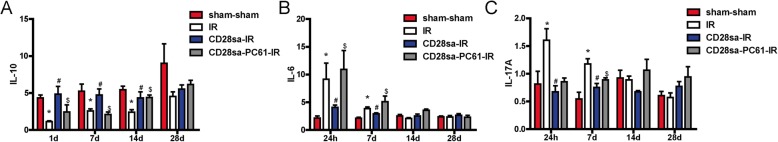
Serum levels of cytokines from mice. **a.** Interleukin-10 expression at different time points after IR injury. **b.** Serum levels of interleukin-6 from different time points after IR injury. **c.** Interleukin-17A expression from different time points after IR injury. ^*^P < 0.05 compared with the sham group (n = 6). ^#^*P* < 0.05 compared with the IR group (n = 6). ^$^*P* < 0.05 compared with the CD28sa-IR group (n = 6)

To further investigate the mechanism of CD28sa-induced anti-inflammation and anti-fibrosis effects, we examined the serum levels of 23 cytokines in mice over time after IRI. The expression of several cytokines differed between the IR group and CD28sa-IR. IL-10, the most well-known Treg effector cytokine, was increased in the CD28sa-IR group compared with the IR group at 24 h and 7 days after IRI (Fig. 6a). IL-6, a pro-inflammatory cytokine, which may associate with downregulated Th17 cells, presented a decrease in the CD28sa-IR group at 24 h and 7 days after IRI (Fig. 6b). IL-17A, the typical effector cytokine of Th17 cells was decreased significantly in the CD28sa-IR group compared with that in the IR-group at 24 h and 7 days (Fig. 6c).

## Discussion

In the present study, we demonstrated the following points: (i) we used the previously reported mouse IRI model and extended the observation window to 7, 14, and 28 days post IRI. A single injection of CD28sa was able to provide remarkable functional and histological protection to the kidneys with less extracellular matrix deposition; (ii) The immuno-inflammatory response of CD28sa pre-treated IRI mice was characterized by an increased percentage of Tregs and MHCII^+^CD11c^+^ dendritic cells, a decreased percentage of Th17 cells and increased secretion of the Treg effector cytokine, IL-10. (iii) CD28sa pretreatment also resulted in less renal cell apoptosis and less oxidative stress marked by less TUNEL and 8-OHdg positive cells. These results demonstrated that CD28sa pretreatment induced a systemic immune tolerance status characterized by expansion of Tregs and CD11c^+^MHCII^+^ dendritic cells, resulting in reduced chronic kidney injury and better long-term prognosis.

CD25^+^Foxp3^+^ Tregs are important in the negative regulation of immune responses and immune tolerance in various injury models such as brain damage after ischemia stroke [19] and chronic destructive arthritis [20]. Sang-Kyung Jo’s team reported that Tregs were likely to contribute to the repair process in IR injury as well as to the tolerance induction mechanism upon subsequent injury [21]. CD28sa amplifies T cell receptor (TCR) signals and is observed to have pronounced therapeutic effects in rodent models for infection-associated inflammation [22], solid-organ transplantation [23–25], rheumatoid arthritis [26] and ischemic organ injury [14, 19]. Moreover, CD28sa-activated Tregs were shown to switch to an IL-10-secreting phenotype and to accumulate at sites of injury [27]. Taken together, it is reasonable to hypothesize that CD28sa exerted a protective effect in the recovery phase of IR by mediating a tolerance induction mechanism. Although, the magnitude of increase in the systemic percentage of Tregs in our study was small from day 7 after IRI, the role of Tregs proved to be important because partial depletion of Tregs using the anti-CD25 antibody, PC61, prophylactically reversed the protective effect of CD28sa. This result was consistent with the protective mechanism of Tregs in the recovery phase of IR mice preconditioned with ischemia [21].

The Treg expansion effect of CD28sa depended largely on the MHCII expression of antigen presenting cells [28]. Therefore, we also tested the percentage of MHCII^+^CD11c^+^ dendritic cells (DCs) from day 7 after IRI injury in mice. Macrophages and DCs are both derived from monocyte/macrophage-lineage common precursor cells and share many surface markers including CD11c. It is thus difficult to distinguish them in an in-vivo system. FCM results showed that the percentage of CD11c^+^MHCII^+^ dendritic cells in kidneys was increased upon CD28sa pretreatment at 7 days after IR injury whereas this increment was abolished by partial Tregs depletion using PC61. This is consistent with a previous report demonstrating that CD11b^+^ cells might represent tolerogenic DCs that potentiate Treg activation [29]. We also observed that cytokine secretion changes in the kidneys post-IR. Among tissue cytokines, IL-6, a well-known inflammatory cytokine, was decreased significantly in CD28sa pre-treated ischemic kidneys at 24 h after IR.

CD8^+^ Tregs have been reported to inhibit effector T cell populations through oxidative phosphorylation in microvesicles [30]. Oxidative stress has also been reported to affect the functions of Tregs [31]. In this study, the effects of CD28sa on apoptosis and oxidative stress in the kidney was also tested at 28 days after IR injury. Prophylactic administration of CD28sa still resulted in less apoptotic kidney cells and ameliorated oxidative stress burden. Combined with the results from our previous study, the beneficial effects of CD28sa are presented in the short-term (within 7 days post IRI), as well as in the long-term (at 28 days post IRI).

It is well known that a severe AKI episode can lead to CKD. Further, sustained inflammation is associated with CKD, our observations in mouse IRI models have revealed a negative association between CD28sa triggered Treg expansion and CKD progression. Renal fibrosis development tended to be much slighter in the CD28sa-IR group than that in the solo IRI. On day 28 post IR, the latter showed a significantly greater Sirius-red-positive area than that in the former, along with prominent intrarenal inflammation, apoptosis, and oxidative stress, suggesting that pretreatment with CD28sa may mitigate renal fibrosis in the long term via upregulation of Tregs.

However, several limitations exist in this study worth mentioning. First, we solely investigated the percentage of Foxp3^+^ Tregs in the progression of CKD following IRI-induced AKI. The lack of a reliable biomarker which was predictive of uncontrolled T cells stimulation by CD28sa was the pivotal factor for the failure of clinical safety testing [32]. We didn’t analyze the expression of CD44 and CD62L in Tregs to distinguish between activated Tregs and resting Tregs. In the future study, we need to focus on related inhibitory signals such as PD-1, which could be used to predict the activation state of Tregs, serving as a potential biomarker to indicate the effects of Treg stimulatory biologic, CD28sa. Secondly, although we had identified the role of CD28sa induced Tregs expansion in retarding AKI to CKD transition, the pharmacodynamics and pharmacokinetics of CD28sa remained unclear. Third, although we have observed the homeostasis between Tregs and Th17 induced by CD28sa, the exact molecular mechanism of Th17 suppression secondary to CD28sa treatment is still unclear. A complete overview of all T-helper cell reactions to IRI or to CD28sa should be required.

## Conclusions

In summary, we provide evidence that CD28sa treatment negatively regulates immune responses in a way mediated by the Th 17 cells /Tregs balance, which might promote post-IRI recovery of the kidney. Identifying the mechanisms of CD28sa induced peripheral immune tolerance might be helpful to develop novel strategies for improving post-IRI prognosis.

## Data Availability

The datasets used and/or analysed during the current study are available from the corresponding author on reasonable request.

## Abbreviations

8-OHdg: 8-Oxoguanine
AKI: Acute kidney injury
APC: Allophycocyanin
CD: Cluster of differentiation
CD28sa: superagonistic CD28
CKD: Chronic kidney disease
DC: Dendritic cells
ECM: Extracellular matrix
ESRD: End stage renal disease
FACS: Fluorescence-activated cell sorting
FITC: Fluorescein isothiocyanate
Foxp3: Forkhead/winged helix transcription factor p3
G-CSF: Granulocyte-colony stimulating factor
G-MCSF: Granulocyte-macrophage colony stimulating factor
IHC: Immunohistochemistry
IL-10: Interleukin-10
IL-12: Interleukin-12
IL-17: Interleukin-17
IL-1α: Interleukin-1a
IL-1β: Interleukin-1β
IL-2: Interleukin-2
IL-3: Interleukin-3
IL-4: Interleukin-4
IL-5: Interleukin-5
IL-9: Interleukin-9
INF-α: Interferon- γ
IR: Ischemia-reperfusion
IRI: Ischemia-reperfusion injury
MCP-1: Monocyte chemotactic protein-1
MHCII: Major histocompatibility complex class II
MIP-1α: Macrophage inflammatory protein-1α
MIP-1β: Macrophage inflammatory protein-1β
NK: Natural killer cells
PBS: Phosphate buffer
PE: Phycoerythrin PAGE polyacrylamide gel electrophoresis
RT: Room temperature
SDS: Sodium dodecyl sulfate
TCR: T cell receptor
Th: T-helper
TNF-α: Tumor necrosis factor-α
Tregs: Regulatory T cells

## References

[1] Fortrie G, de Geus HRH, Betjes MGH. The aftermath of acute kidney injury: a narrative review of long-term mortality and renal function. Crit Care (London, England). 2019;23:24. 10.1186/s13054-019-2314-z PMC6346585 30678696

[2] Bellomo R, Kellum JA, Ronco C (2012). Acute kidney injury. Lancet.

[3] Fang Y, Teng J, Ding X (2015). Acute kidney injury in China. Hemodialysis international International Symposium on Home Hemodialysis.

[4] Hsu RK, McCulloch CE, Dudley RA, Lo LJ, Hsu CY (2013). Temporal changes in incidence of dialysis-requiring AKI. Journal of the American Society of Nephrology : JASN.

[5] Gandolfo MT, Jang HR, Bagnasco SM, Ko GJ, Agreda P, Satpute SR, Crow MT, King LS, Rabb H (2009). Foxp3+ regulatory T cells participate in repair of ischemic acute kidney injury. Kidney Int.

[6] Jiang SH, Liu CF, Zhang XL, Xu XH, Zou JZ, Fang Y, Ding XQ (2007). Renal protection by delayed ischaemic preconditioning is associated with inhibition of the inflammatory response and NF-kappaB activation. Cell Biochem Funct.

[7] Zhang Bing-Ying, Fang Yi, Jiao Xiao-Yan, Wu Sheng, Cai Jie-Ru, Zou Jian-Zhou, Ding Xiao-Qiang (2016). Delayed ischaemic preconditioning in the presence of galectin-9 protects against renal ischaemic injury through a regulatory T-cell dependent mechanism. Nephrology.

[8] Cao CC, Ding XQ, Ou ZL, Liu CF, Li P, Wang L, Zhu CF (2004). In vivo transfection of NF-kappaB decoy oligodeoxynucleotides attenuate renal ischemia/reperfusion injury in rats. Kidney Int.

[9] Lee Jae Wook, Bae Eunjin, Kwon Sun-Ho, Yu Mi-Yeon, Cha Ran-Hui, Lee Hajeong, Kim Dong Ki, Lee Jung Pyo, Ye Sang-Kyu, Yoo Joo-Yeon, Park Dong Jun, Kim Yon Su, Yang Seung Hee (2018). Transcriptional modulation of the T helper 17/interleukin 17 axis ameliorates renal ischemia-reperfusion injury. Nephrology Dialysis Transplantation.

[10] Sakai Kenji, Nozaki Yuji, Murao Yoshinori, Yano Tomohiro, Ri Jinhai, Niki Kaoru, Kinoshita Koji, Funauchi Masanori, Matsumura Itaru (2019). Protective effect and mechanism of IL-10 on renal ischemia–reperfusion injury. Laboratory Investigation.

[11] Jiang S, Chen Y, Zou J, Xu X, Zhang X, Liu C, Fang Y, Ding X (2009). Diverse effects of ischemic pretreatments on the long-term renal damage induced by ischemia-reperfusion. Am J Nephrol.

[12] Yan JJ, Lee JG, Jang JY, Koo TY, Ahn C, Yang J (2017). IL-2/anti-IL-2 complexes ameliorate lupus nephritis by expansion of CD4(+)CD25(+)Foxp3(+) regulatory T cells. Kidney Int.

[13] Bai M, Zhang L, Fu B, Bai J, Zhang Y, Cai G, Bai X, Feng Z, Sun S, Chen X (2018). IL-17A improves the efficacy of mesenchymal stem cells in ischemic-reperfusion renal injury by increasing Treg percentages by the COX-2/PGE2 pathway. Kidney Int.

[14] Liang Y, Li Y, Kuang Q, Ding X, Wei Z, Fang Y (2017). Superagonistic CD28 protects against renal ischemic injury by expansion of regulatory T-cell. Am J Nephrol.

[15] Yuan Y, Chen Y, Zhou Z, Jiao Y, Li C, Zheng Y, Lin Y, Xiao J, Chen Z, Cao P (2018). Association between chronic inflammation and latent infection of Propionibacterium acnes in non-pyogenic degenerated intervertebral discs: a pilot study. Eur Spine J.

[16] Cunningham MW, Vaka VR, McMaster K, Ibrahim T, Cornelius DC, Amaral L, Campbell N, Wallukat G, McDuffy S, Usry N, Dechend R, LaMarca B (2019). Renal natural killer cell activation and mitochondrial oxidative stress; new mechanisms in AT1-AA mediated hypertensive pregnancy. Pregnancy hypertension.

[17] Maj T, Wang W, Crespo J, Zhang H, Wang W, Wei S, Zhao L, Vatan L, Shao I, Szeliga W, Lyssiotis C, Liu JR, Kryczek I, Zou W (2017). Oxidative stress controls regulatory T cell apoptosis and suppressor activity and PD-L1-blockade resistance in tumor. Nat Immunol.

[18] Djudjaj S, Martin IV, Buhl EM, Nothofer NJ, Leng L, Piecychna M, Floege J, Bernhagen J, Bucala R, Boor P (2017). Macrophage migration inhibitory factor limits renal inflammation and fibrosis by counteracting tubular cell cycle arrest. Journal of the American Society of Nephrology : JASN.

[19] Na SY, Mracsko E, Liesz A, Hunig T, Veltkamp R (2015). Amplification of regulatory T cells using a CD28 superagonist reduces brain damage after ischemic stroke in mice. Stroke.

[20] Win SJ, Kuhl AA, Sparwasser T, Hunig T, Kamradt T (2016). In vivo activation of Treg cells with a CD28 superagonist prevents and ameliorates chronic destructive arthritis in mice. Eur J Immunol.

[21] Cho WY, Choi HM, Lee SY, Kim MG, Kim HK, Jo SK (2010). The role of Tregs and CD11c(+) macrophages/dendritic cells in ischemic preconditioning of the kidney. Kidney Int.

[22] Guilliams M, Bosschaerts T, Herin M, Hunig T, Loi P, Flamand V, De Baetselier P, Beschin A (2008). Experimental expansion of the regulatory T cell population increases resistance to African trypanosomiasis. J Infect Dis.

[23] Azuma H, Isaka Y, Li X, Hunig T, Sakamoto T, Nohmi H, Takabatake Y, Mizui M, Kitazawa Y, Ichimaru N, Ibuki N, Ubai T, Inamoto T, Katsuoka Y, Takahara S (2008). Superagonistic CD28 antibody induces donor-specific tolerance in rat renal allografts. Am J Transplant.

[24] Kitazawa Y, Fujino M, Sakai T, Azuma H, Kimura H, Isaka Y, Takahara S, Hunig T, Abe R, Li XK (2008). Foxp3-expressing regulatory T cells expanded with CD28 superagonist antibody can prevent rat cardiac allograft rejection. J Heart Lung Transplant.

[25] Urakami H, Ostanin DV, Hunig T, Grisham MB (2006). Combination of donor-specific blood transfusion with anti-CD28 antibody synergizes to prolong graft survival in rat liver transplantation. Transplant Proc.

[26] Beyersdorf N, Gaupp S, Balbach K, Schmidt J, Toyka KV, Lin CH, Hanke T, Hunig T, Kerkau T, Gold R (2005). Selective targeting of regulatory T cells with CD28 superagonists allows effective therapy of experimental autoimmune encephalomyelitis. J Exp Med.

[27] Langenhorst D, Gogishvili T, Ribechini E, Kneitz S, McPherson K, Lutz MB, Hunig T (2012). Sequential induction of effector function, tissue migration and cell death during polyclonal activation of mouse regulatory T-cells. PLoS One.

[28] Langenhorst D, Tabares P, Gulde T, Becklund BR, Berr S, Surh CD, Beyersdorf N, Hunig T (2017). Self-recognition sensitizes mouse and human regulatory T cells to low-dose CD28 Superagonist stimulation. Front Immunol.

[29] Offner H, Vandenbark AA, Hurn PD (2009). Effect of experimental stroke on peripheral immunity: CNS ischemia induces profound immunosuppression. Neuroscience.

[30] Berger CT, Hess C (2016). Neglected for too long? - CD8+ Tregs release NOX2-loaded vesicles to inhibit CD4+ T cells. J Clin Invest.

[31] Anupam K, Kaushal J, Prabhakar N, Bhatnagar A (2018). Effect of redox status of peripheral blood on immune signature of circulating regulatory and cytotoxic T cells in streptozotocin induced rodent model of type I diabetes. Immunobiology.

[32] Thaventhiran Thilipan, Alhumeed Naif, Yeang Han XA, Sethu Swaminathan, Downey Jocelyn S, Alghanem Ahmad F, Olayanju Adedamola, Smith Emma L, Cross Michael J, Webb Steven D, Williams Dominic P, Bristow Adrian, Ball Christina, Stebbings Richard, Sathish Jean G (2014). Failure to upregulate cell surface PD-1 is associated with dysregulated stimulation of T cells by TGN1412-like CD28 superagonist. mAbs.

